# Large left varicocele in a patient with portal hypertension treated via transjugular intrahepatic portosystemic shunt placement and both variceal and varicocele embolization

**DOI:** 10.1186/s12894-023-01268-9

**Published:** 2023-05-15

**Authors:** Ayah Megahed, Todd Schlachter, Joshua Cornman-Homonoff

**Affiliations:** 1 Department of Radiology, Yale New Haven Health, Bridgeport Hospital, Bridgeport, USA; 2 Division of Interventional Radiology, Department of Radiology and Biomedical Imaging, Yale School of Medicine, SP2-213, 20 York Street, New Haven, CT 06510 USA

**Keywords:** Varicocele, Portal hypertension, Cirrhosis, Portosystemic shunt

## Abstract

**Background:**

Scrotal swelling from varicocele is a common complaint in adult men. Varicocele due to portosystemic collaterals is a rare presentation of portal hypertension. Imaging workup and intervention for varicocele in this case is more complex than varicocele due to absent or incompetent valves in the testicular veins and pampiniform plexus.

**Case presentation:**

We present the case of a 53-year-old man with alcohol-related cirrhosis presented with persistent left scrotal heaviness, pain, and swelling found to have a large left varicocele. Given his history of cirrhosis, a contrast-enhanced CT of the abdomen and pelvis was obtained showing that the varices were supplied by a vessel arising from the splenic vein and draining into the left renal vein as well as gastric varices. Varicocele embolization alone is not sufficient in this case, and we treated with transjugular intrahepatic portosystemic shunt, variceal and varicocele embolization.

**Conclusion:**

In patients presenting with a varicocele with a history of cirrhosis/portal hypertension, cross sectional imaging of the abdomen and pelvis should be obtained prior to treatment to evaluate for the presence of varices which may be pressured by varicocele embolization. If present, consideration should be given to referral to an interventional radiologist for possible concurrent variceal embolization and TIPS placement.

## Introduction

A varicocele is an abnormal dilatation of the pampiniform plexus resulting from incompetent venous valves and dependent pooling of blood [1]. Varicoceles are present in the 15% of all males and 30–40% of those with primary infertility; 80–90% are left-sided [2, 3]. Treatment options include open surgery, microsurgical varicocelectomy, and endovascular embolization, any of which may be performed for symptom relief or treatment of infertility [4]. Varicocele development has rarely been reported as a consequence of portal hypertension and may necessitate an alternative management approach [2]. Here we report a case of a symptomatic left varicocele in a patient with portal hypertension and non-bleeding gastric varices managed via transjugular intrahepatic portosystemic shunt placement and embolization of both the varices and varicocele.

## Case report

**Fig. 1:**
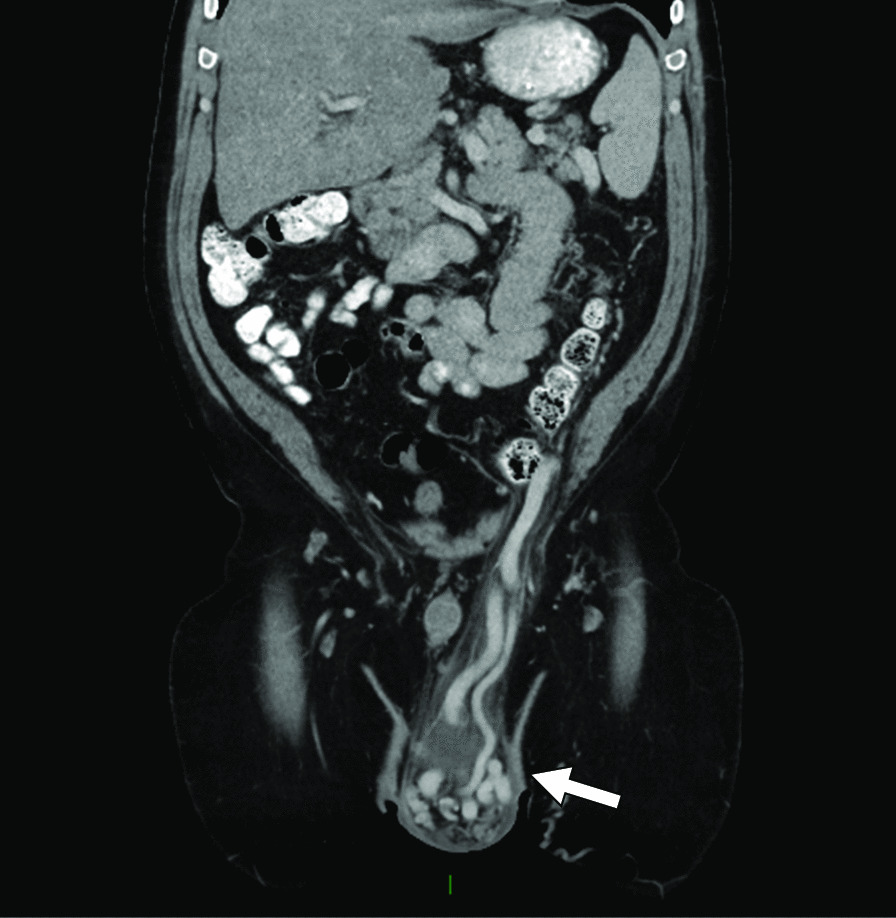
Coronal contrast enhanced CT image showing dilated left spermatic veins (arrow)

**Fig. 2:**
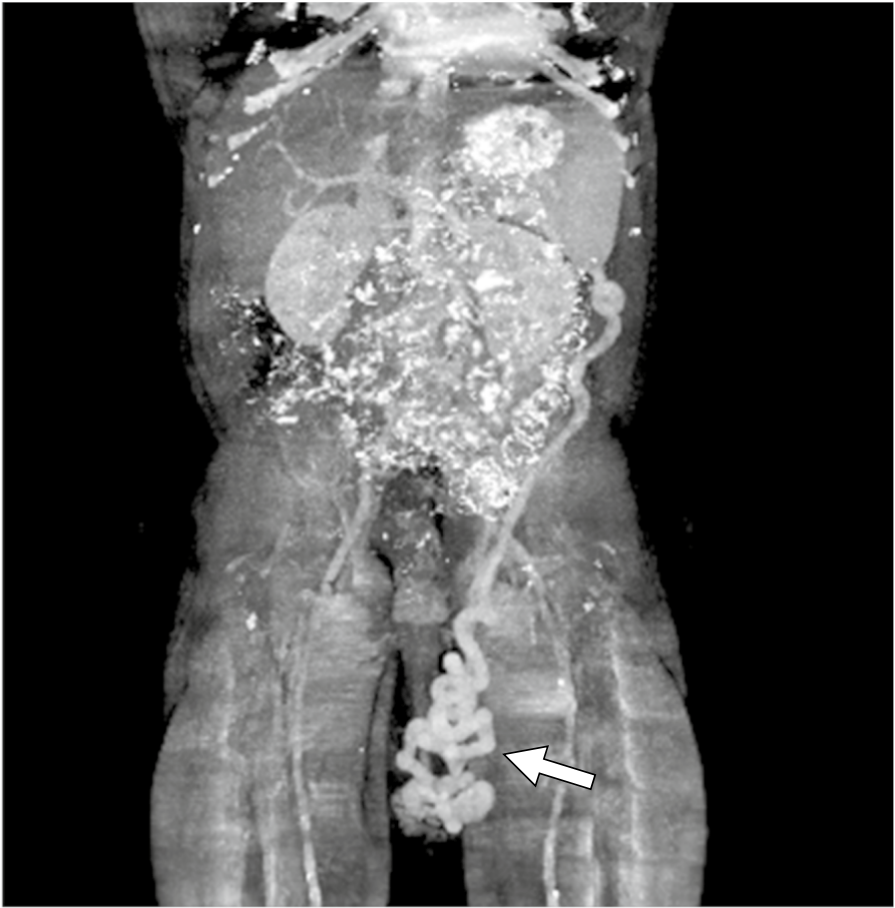
Maximum intensity projection of the prior image showing the dilated left spermatic veins (arrow) arising from the splenic vein and draining into the left renal vein

**Fig. 3:**
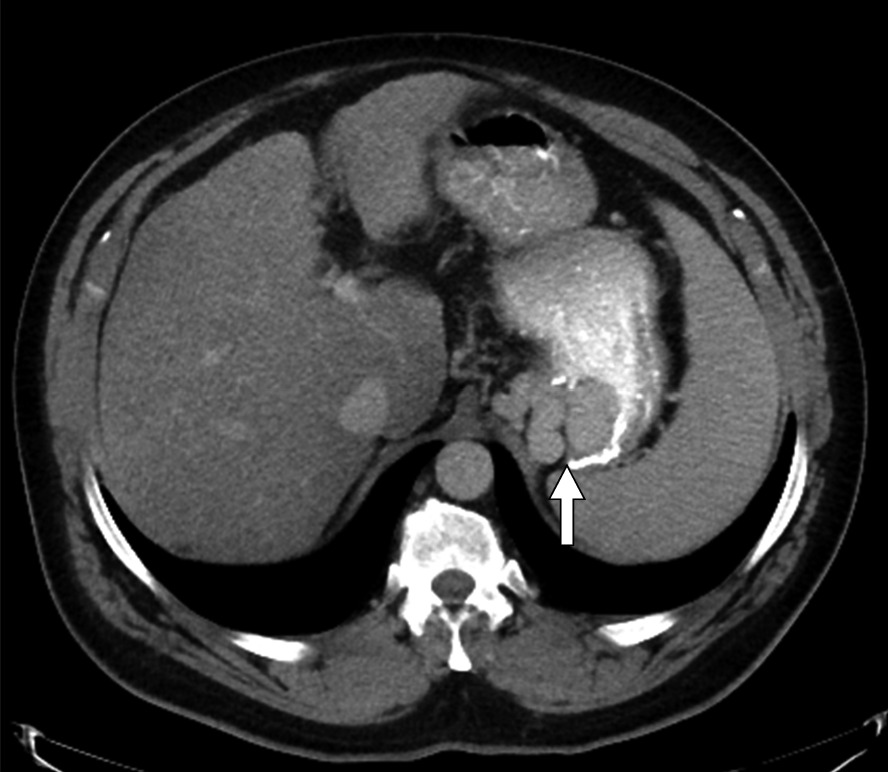
Axial contrast enhanced CT image showing dilated gastric varices (arrow)

A 53-year-old man with alcohol use disorder (abstinent for six months) and alcohol-related cirrhosis presented to an outside hospital with persistent left scrotal heaviness, pain, and swelling and was found on physical examination and ultrasound to have a large left varicocele. Given his history of cirrhosis, a contrast-enhanced CT of the abdomen and pelvis was obtained showing that the varices were supplied by a vessel arising from the splenic vein and draining into the left renal vein (Figs. 1, 2). Stigmata of cirrhosis and portal hypertension were also present, including splenomegaly and large gastric varices (Fig. 3). Labs were notable for MELD 16/MELD-Na 16, calculated from sodium 138 mmol/L, creatinine 0.78 mg/dl, total bilirubin 2.71 mg/dl, and INR 1.7. Given his symptomatology, he was referred for varicocele embolization.

Because there was reversal of flow in the portal and splenic veins such that the varicocele provided most of the portal outflow, isolated embolization would pressurize the gastric varices and possibly precipitate an upper gastrointestinal bleed. Thus, concurrent gastric variceal embolization was also planned. However, because both shunts were functioning as pathways for portal decompression, closing them could result in portal vein thrombosis. Consequently, transjugular intrahepatic portosystemic shunt (TIPS) placement was also planned to provide venous outflow.

**Fig. 4:**
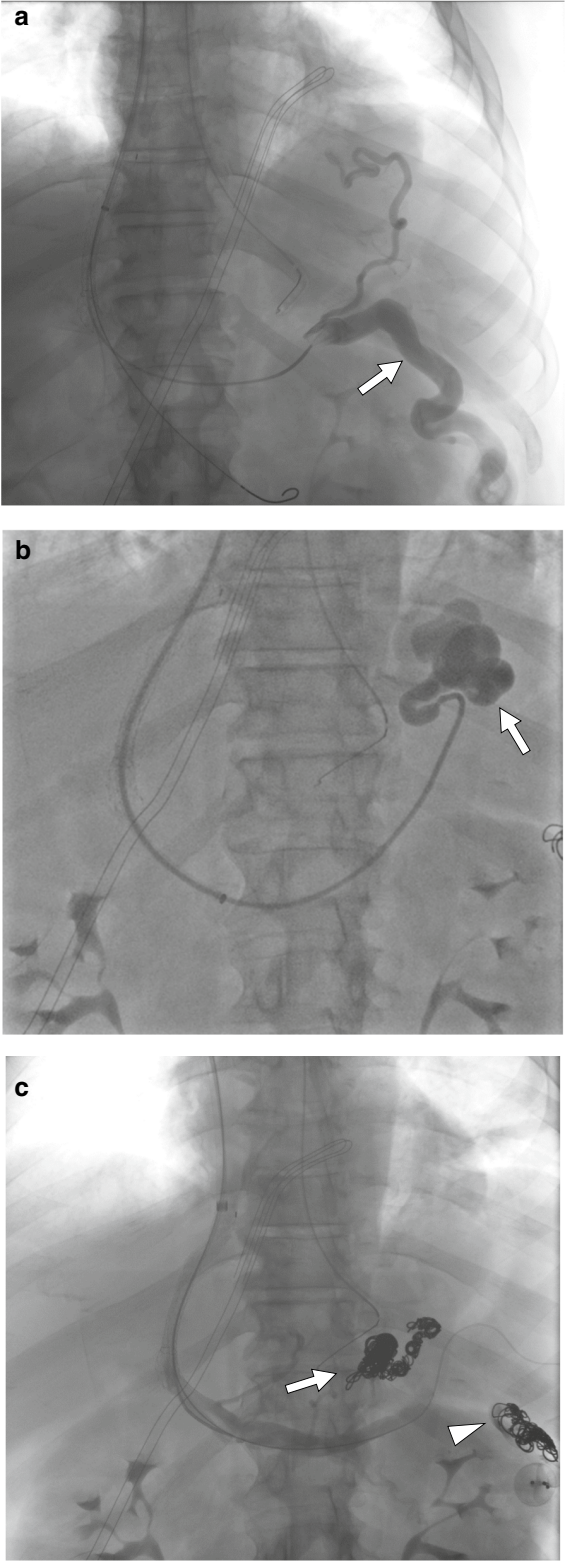
**a** Post TIPS angiogram showing a dilated left testicular vein arising from the splenic vein (arrow). **b** Post TIPS angiogram showing gastric varices (arrow). **c** Post TIPS and embolization angiogram showing successful embolization of the gastric varices (arrow) and varicocele (arrowhead)

Access was obtained in the right internal jugular vein and right atrial, free hepatic vein, and wedged hepatic vein pressures measured. A Rosch-Uchida set (Cook Medical, Bloomington, IN) was subsequently used to obtain transhepatic access to the portal vein and a TIPS placed using a Gore Viator endograft (Gore Medical, Newark, DE) and dilated to 6 mm. The varicocele (Fig. 4a) was then embolized using a combination of plugs and coils, after which the gastric varix (Fig. 4b) was embolized with coils. Final imaging confirmed antegrade flow in the portal vein and TIPS without opacification of the varicocele or gastric varices (Fig. 4c).

The patient experienced worsening scrotal pain on post procedure day 1, which was controlled with non-steroidal anti-inflammatory drugs. He was discharged on post procedure day 2. As of follow up one month later, he was asymptomatic and had experienced no encephalopathy.

## Discussion

A varicocele is a common finding in both symptomatic and asymptomatic men and warrants treatment when causing infertility or discomfort. The normal venous drainage of the testicle is via the pampiniform plexus, which coalesces into the testicular vein before draining into the renal vein on the left and IVC on the right [5]. Although the present case demonstrated conventional anatomy, the added inflow from the splenorenal shunt likely pressurized the renal and testicular veins, resulting in retrograde flow in the latter.

Physical examination and Doppler ultrasound are usually adequate for diagnosis [6]. However, this evaluation can miss relevant findings in patients with underlying portal hypertension. Due to the rarity of this condition, there is no consensus on evaluation or treatment. However, because isolated clipping and/or embolization of the varicocele may result in pressurization of existing varices, it could precipitate life-threatening hemorrhage. Similarly, concurrent embolization of both the varicocele and varices may adversely affect portal outflow and lead to portal vein thrombosis, which similarly may necessitate urgent intervention or preclude transplant candidacy. Consequently, the authors believe that in patients presenting with varicocele with a history of cirrhosis, a contrast-enhanced CT or MR of the abdomen and pelvis should be obtained prior to intervention.

## Conclusion

In patients presenting with a varicocele with a history of cirrhosis/portal hypertension, cross sectional imaging of the abdomen and pelvis should be obtained prior to treatment to evaluate for the presence of varices which may be pressured by varicocele embolization. If present, consideration should be given to referral to an interventional radiologist for possible concurrent.

## Data Availability

Not Applicable (case report).

## Abbreviations

CT: Computed tomography
MELD score: Model for end-stage liver disease
TIPS: Transjugular intrahepatic portosystemic shunt
